# Books and Magazines

**Published:** 1909-07

**Authors:** 


### Books and Magazines.

VACCINE AND SERUM THERAPY—Including also a study of Infections, Theories of Immunity, Opsonins and the Opsonic Index. By Edwin Henry Schorer, B. S., M. D., Assistant Professor of Parasitology and Hygiene, University of Missouri; formerly Assistant at Rockefellow Institute for Medical Research, New York. Illustrated. St. Louis, 1909. C. V. Mosby Co. Large 8vo, cloth, 125 pages. Price not stated.

Every practicing physician who would keep up with the progress oi medical science should study this book. It is the latest. Most doctors have but a vague idea of immunity, and a still vaguer one, or none, of what is meant by the opsonic theory, the opsonic index, etc. And such ignorance is inexcusable, inasmuch as such clear descriptions as are given in Dr. Schorer's book are available. Chapter III is devoted to the opsonic index, a valuable aid to diagnosis and treatment. Chapter II deals with immunity, and gives several theories of how it is brought about, Erlich's side chain theory being the principal and most generally accepted; the opsonin theory,

Metchnikoff's well-known phagocytic theory, etc. The book, of course, is very technical.

Get the book, Doctor, and read all about it. Our Dr. J. W. McLaughlin, late Professor of Medicine in the University of Texas Medical School, now Regent of the University (Board) and President of the Texas Academy of Science, has a theory of immunity in which he opposes the side chain theory and controverts Erlich's arguments. This paper can be had in pamphlet form from the author, at Austin.

---

Hand Book of Diseases of the Rectum. By Louis J. Hirschman, M. D., Detroit, Mich., Lecturer on Rectal Surgery and Clinical Professor of Proctology, Detroit College of Medicine, etc. 147 illustrations, mostly original; two-colored plates. St. Louis, 1909. C. V. Mosby Book and Pub. Co. Cloth, 363 pages. Price, ——.

This is a splendid book and most timely. It "fills a long-felt want." Nothing on the subject has reached us since Matthews' book some ten years ago. If there is any one subject that the average physician knows less about than another it is diseases of the rectum. It is notorious that the subject is not taught in the schools in a way in keeping with its importance, a few lectures during the term being given as part of the general course. Hence the smaller fellows being unable to handle rectal diseases—even fistula and piles—are glad to send cases to the city specialist, who reaps a rich reward. For one with pruritus ani, or piles, or fistula, will give all he possesses, if necessary, to get rid of it. I recommend my readers to get this book. It is a treasure trove.

---

Practical Medicine Series. 1909. Vol. 3. Eye, Ear, Nose and Throat. Wood-Andrews-Head, the Year Book Publishers, Chicago. The set of 10 volumes is sold for $10. Separate volumes, $1.25. The volume before us is uniform with the series published any year. Cloth, 350 pages.

Its contents, as usual, are made up from gleanings from current medical literature, and the books are, therefore, a year or more ahead of the text-books.

---

Practical Dietetics—A Text-book for Nurses. Fifth edition just out. By Alida Frances Pattee, Mt. Vernon, N. Y. A. F. Pattee, Publisher and Bookseller. $1.00; postage, 10c. 12mo. Cloth, 300 pages.

This valuable little book has been issued each year, reaching now its fifth edition. Each edition has been noticed in these pages. We will only add the publisher's statement, that the "Annual issue of Practical Dietetics consists of a 10,000 edition. The book has been adopted by many special mediums; it is recommended by all the State boards of examiners of nurses that have thus far been appointed; adopted for the United States and Canadian governments for use in medical department of the army; added to the authorized text-book list of the Boston and New York public schools; used in the leading medical colleges and hospitals, training schools, etc."

---

THE PRESIDENT'S HOMES COMMISSION SERIES: We have received the following named publications, issued by the government, published by the Commission:

Report of Committee on Social Betterment, by Geo. M. Kober, M. D., LL. D, chairman committee. Paper, 280 pages.

Report of Committee on Improvement of Existing Homes and the Elimination of Unsanitary and Alley Houses, by Wm. H. Baldwin, chairman committee.

Report of Committee on Building of Model Houses, by General G. M. Sternberg, M. D., LL. D., chairman committee.

Industrial and Personal Hygiene, by Geo. M. Kober, M. D., LL. D.

Part of report of Commission on Social Betterment, etc.

These books may be had free from either of the authors named.

---

THE ARTERIES OF THE GASTRO-INTESTINAL TRACT, WITH INOSCULATION CIRCLE. By Byron Robinson. Everybody knows him. In addition to the plates, in the text is given a full description, embracing the anatomy and physiology, with application for treatment.

In a note on the title page the author says: "The function of the inosculation circle is to congest its peripheral viscus and to transport blood volume from one viscus to another." A good plate if the inosculation circle is given. Other plates are: Concentric gastric circles, ileo-colic circle, entero-colic circle, gastro-entero-colic circle, pancreatic circle, ilco-colic arches.

This book is worth many times the small price at which it is sold, $1.50, and will give one a better idea of the anatomy and relations of the arteries than any text-book I know.

---

CONFESSIONS OF A NEURASTHENIC. By William Taylor Marrs, M. D. Illustrated with original and appropriate drawings. Published in a neat 12mo volume of 115 pages. Bound in extra cloth. Price, $1.00 net. F. A. Davis Co., Publishers, 1914-16 Cherry Street, Philadelphia, Pa.

This is a graphic and intensely entertaining, as well as instructive account of the ups and downs, with the incidental variations in the career of one of those numerous individuals whose complex and highly developed nervous organization has sounded the depths and scaled the heights of untold pain and pleasure, pathos and humor.

Modern Medicine; Its Theory and Practice—In Original Contributions by American and Foreign Authors. Edited by William Osler, M. D., Regius Professor of Medicine in Oxford University, England; formerly Professor of Medicine in Johns Hopkins University, Baltimore; in the University of Pennsylvania, Philadelphia, and in McGill University, Montreal. Assisted by Thomas McCrea, M. D., Associate Professor of Medicine and Clinical Therapeutics in Johns Hopkins University, Baltimore. In seven octavo volumes of about 900 pages each, illustrated. Volume VI, Diseases of the Urinary System, of the Ductless Glands, of the Muscles, Diseases of Obscure Causation, Vasomotor and Trophic Disorders, Medical Aspects of Life Insurance. Just ready. Price per volume: cloth, $6.00 net; leather, $7.00 net; half morocco, $7.50 net. Lea & Febiger, Publishers, Philadelphia and New York, 1909.

The sixth volume of Osler's *Modern Medicine,* just off press, covers a very wide and important range of subjects, namely, the diseases of the urinary system, of the ductless glands, of the muscles, those of obscure causation, vasamotor and trophic disorders, and the medical aspects of life insurance. These diseases are all handled by specially competent men. John McCrae of Toronto begins the volume with two chapters on the kidney, followed by two on urinary anomalies and uraemia by Garrod of London. Herrick of Chicago deals with all aspects of nephritis, as well as amyloid disease, and Thomas R. Brown of Baltimore considers pyogenic and tubercular affections of the kidney. Its medico surgical aspects, from the pen of H. H. Young of Baltimore, conclude this section. George Dock, formerly of Ann Arbor, and now of New Orleans, has written the entire section on the ductless glands. Longcope of Philadelphia considers Hodgkin's Disease; T. McCrae of Baltimore, arthritis deformans; Dock of New Orleans, osteomalacia, and D. J. McCarthy of Philadelphia, astasia-abasia and adiposis dolorosa. Together with W. R. Steiner of Hartford, McCarthy has written the section on muscular diseases. The editor, Dr. Osler, with his former colleague, C. P. Emerson of Baltimore, handles the section on vasomotor and trophic disorders, and Charles Lyman Greene of St. Paul concludes with the medical aspects of life insurance.

It is obvious from the foregoing brief of contents that the English-speaking world of medicine is ably and impartially represented, and that the cosmopolitanism, which is a distinguishing feature of *Modern Medicine* is constantly maintained. The best collective medical knowledge of the world is being placed at command of every practitioner in the most helpful form. The seventh volume will cover diseases of the nervous system, and will complete this great library of medicine. Its practical value is attested by its phenomenal success.

---

Writing the Short Story. By J. Berg Esenwein, A. M., Lit. D. Editor of Lippincott's Monthly Magazine. Author of "How to Attract and Hold an Audience." A helpful book for writers. Cloth. 12mo. 488 pages. Price, $1.25. Published by Hinds, Noble & Eldredge, New York.

The population of the United States seems to be composed mostly of short-story writers—and not mostly fools, as Carlyle said of the population of the British Isles. If all these writers would "read, mark and inwardly digest" Dr. J. Berk Esenwein's remarkable new volume on "Writing the Short-Story," the public would be grateful, for the quality of magazine and newspaper fiction would doubtless improve—and it certainly needs bettering.

"Writing the Short Story" is a substantial and handsome volume of 450 pages, chock-a-block full of sensible and helpful ideas for those who write and, indeed, also for those who would intelligently read, the short story. Its chapters on gathering literary materials, and the structure of the plot are clear and suggestive, while the treatment of dialogue, characters, titles, and opening and closing the story must prove valuable to experienced writers as well as to beginners. The trouble with previous books on this subject has been that they have been written by theorists who rarely if ever bought or sold a short story in their lives. This volume is different. It is the work of a practiced writer, and an editor who has handled many thousands of manuscripts from writers great and small. Therefore, his advice on how to prepare manuscript, how to sell the story, and all the practical end of the matter, is quite as valuable as his captivating chapters on the technique of the short story. Every writer, young or old, will find in "Writing the Short Story" a well-spring of inspiration.

